# Benefits of Hypothermia for Young Patients with Acute Subdural Hematoma: A Computed Tomography Analysis of the Brain Hypothermia Study

**DOI:** 10.1089/neur.2021.0080

**Published:** 2022-07-15

**Authors:** Hitoshi Kobata, Yasuhiro Kuroda, Eiichi Suehiro, Tadashi Kaneko, Motoki Fujita, Naofumi Bunya, Kei Miyata, Akihiko Inoue, Toru Hifumi, Yasutaka Oda, Kenji Dohi, Susumu Yamashita, Tsuyoshi Maekawa

**Affiliations:** ^1^ Department of Neurosurgery, Osaka Mishima Emergency Critical Care Center, Takatsuki, Japan.; ^2^ Department of Emergency, Disaster, and Critical Care Medicine, Kagawa University School of Medicine, Takamatsu, Japan.; ^3^ Department of Neurosurgery, International University of Health and Welfare, School of Medicine, Narita, Japan.; ^4^ Emergency and Critical Care Center, Mie University Hospital, Tsu, Japan.; ^5^ Advanced Medical Emergency and Critical Care Center, Yamaguchi University Hospital, Ube, Japan.; ^6^ Department of Emergency Medicine and Sapporo Medical University, Sapporo, Japan.; ^7^ Neurosurgery, Sapporo Medical University, Sapporo, Japan.; ^8^ Department of Emergency and Critical Care Medicine, Hyogo Emergency Medical Center, Kobe, Japan.; ^9^ Department of Emergency and Critical Care Medicine, St. Luke's International Hospital, Tokyo, Japan.; ^10^ Department of Emergency, Disaster and Critical Care Medicine, Showa University, Tokyo, Japan.; ^11^ Emergency and Critical Care Center, Tokuyama Central Hospital, Tokuyama, Japan.; ^12^ Yamaguchi Prefectural University, Yamaguchi, Japan.

**Keywords:** acute subdural hematoma, computed tomography, evacuated hematoma, hypothermia, targeted temperature management, traumatic brain injury

## Abstract

Therapeutic hypothermia for severe traumatic brain injury (TBI) has been repeatedly studied, but no past studies have assessed the detailed head computed tomography (CT) findings. We sought to investigate individual CT findings of severe TBI patients treated with targeted temperature management utilizing the head CT database obtained from the Brain Hypothermia study. Enrolled patients underwent either mild therapeutic hypothermia (32.0°C−34.0°C) or fever control (35.5°C−37.0°C). We assessed individual head CT images on arrival and after rewarming and investigated the correlations with outcomes. The initial CT data were available for 125 patients (hypothermia group = 80, fever control group = 45). Baseline characteristics and CT findings, such as hematoma thickness and midline shift, were similar in all aspects between the two groups. The favorable outcomes in the hypothermia and fever control groups were 38 (47.5%) and 24 (53.3%; *p* = 0.53) for all 125 patients, respectively; 21 (46.7%) vs. 10 (38.5%; *p* = 0.50) for 71 patients with acute subdural hematoma (SDH), respectively; and 12 (75.0%) vs. 4 (36.4%; *p* = 0.045) in 27 young adults (≤50 years) with acute SDH, respectively. There was a trend toward favorable outcomes for earlier time to reach 35.5°C (190 vs. 377 min, *p* = 0.052) and surgery (155 vs. 180 min, *p* = 0.096) in young patients with acute SDH. The second CT image revealed progression of the brain injury. This study demonstrated the potential benefits of early hypothermia in young patients with acute SDH, despite no difference in CT findings between the two groups. However, the small number of cases involved hindered the drawing of definitive conclusions. Future studies are warranted to validate the results.

## Introduction

Therapeutic hypothermia has been clinically applied to severe traumatic brain injury (TBI) with enthusiasm during the 1990s.^1–3^ However, large randomized controlled trials (RCTs) since the 2000s have failed to show any benefit.^4–8^ Although numerous experimental studies have reported that lowering post-traumatic temperature attenuated multiple secondary injury mechanisms, including excitotoxicity, free radical generation, apoptotic cell death, and inflammation,^9^ these promising effects of hypothermia did not improve outcomes in clinical practice.^10^ Benefits have not been confirmed both in the early induction of hypothermia aimed at mitigating detrimental cascades after trauma^4–6,8^ and in late-rescue hypothermia for elevated intracranial pressure (ICP).^7^ A meta-analysis of high-quality RCTs confirmed the lack of benefits.^11^ In contrast, another meta-analysis showed that hypothermia was only beneficial if the cooling index—calculated from targeted cooling temperature, cooling duration, and rewarming speed—was sufficiently high.^12^

TBI is a heterogeneous, extremely complicated clinical condition that affects the most complex organ of the body, the brain. Unlike the relatively uniform whole-brain ischemia caused by cardiac arrest, the damage arising from TBI is highly complex and individualized. The heterogeneity of TBI is one of the essential factors for evaluating the effects of hypothermia. Although the current Brain Trauma Foundation TBI Guidelines do not recommend early, short-term (48 h post-injury) prophylactic hypothermia for diffuse injury,^13^ potential benefits of hypothermia have been shown in young adults with evacuated mass lesions when early surgical interventions were conducted.^14,15^

In previous studies on hypothermia, head computed tomography (CT) findings were categorized according to the Traumatic Coma Data Bank (TCDB) classification^4–6,8,15^; however, the types, nature, and extent of evacuated mass lesions have not been investigated. Therefore, we aimed to re-evaluate the significance of hypothermia, if any, through detailed CT studies using the Brain Hypothermia (B-HYPO) study database.

## Methods

### Study design

This was a *post hoc* analysis utilizing the head CT database from the B-HYPO study, a multi-center RCT conducted between 2002 and 2008 in Japan.^6^ The B-HYPO study aimed at rapid and prolonged targeted temperature management (TTM) for brain protection. The institutional review boards of the participating hospitals approved the protocol and was registered with the University Hospital Medical Information Network (UMIN) site in Japan (UMIN-CTR no.: C000000231) and the National Institutes of Health (Clinical-Trials.gov identifier: NCT00134472) in the United States.

We recruited TBI patients 15–69 years of age with Glasgow Coma Scale (GCS) scores of 4–8 and who could undergo cooling within 2 h after injury. Patients without abnormal CT findings or those with epidural hematomas were excluded. Patients were categorized into either a mild hypothermia group (TTM at 32.0°C–34.0°C) or a fever control group (TTM at 35.5°C–37.0°C) at a ratio of 2:1, using a list generated by the UMIN computer system. If informed consent could not be obtained within 2 h of admission, the consent policy was waived.

### Monitoring and management

We attempted to achieve the target temperature within 6 h of TBI onset and maintain it for ≥72 h with ICP monitoring. Cooling blankets, rapid cold fluid infusion, and/or cold gastric lavage were used to induce TTM. The temperature of the internal jugular vein was monitored and controlled using conventional surface cooling methods. Hemodynamic status was strictly monitored using a pulmonary arterial catheter. Patients in the hypothermia group were rewarmed at a rate of <1°C per day. Core body temperature was maintained at <38°C for 7 days after onset of TBI in both groups.

### Data collection and analysis

Except the original head CT images, all data were transmitted to the UMIN center by an internet-based system. In addition to individual factors, the International Mission for Prognosis and Analysis of Clinical Trials (IMPACT) score^16^ was calculated to determine severity of the injury. In addition, the core model (age, GCS motor score, and pupillary reactivity), extended model (core model plus hypoxia, hypotension, the TCDB CT classification,^17^ traumatic subarachnoid hemorrhage [tSAH], and acute epidural hematoma [EDH]), and laboratory model (extended model plus glucose and hemoglobin) were analyzed.

### Head computed tomography assessment

Head CT scans were performed on admission and after rewarming around day 7 per protocol. Researchers in each participating hospital assessed the presence or absence of tSAH, cerebral contusion, acute subdural hematoma (SDH), acute EDH, intraventricular hematoma (IVH), diffuse axonal injury (DAI), and any other findings. If multiple lesions were present, all lesions were mentioned. Assessments were uploaded and categorized according to TCDB classification. Further, the original CT images were recorded on electronic media, sent to the representative researcher, and saved in the database.

Using the head CT database, two authors (H.K. and Y.K.) independently evaluated the following individual CT findings: 1) the status of basal cisterns; 2) presence of tSAH; 3) presence and degree of midline shift; 4) presence and type of intracranial lesions (SDH, EDH, contusion, and IVH); 5) hematoma thickness; and 6) lesion laterality. The Rotterdam CT score (Supplementary Table S1)^18^ was also calculated. Clinical data and outcomes were concealed when reviewing the images.

### Outcome measures

The Glasgow Outcome Scale score was assessed by blinded assessors after 6 months. Outcomes were dichotomized as “favorable” (good recovery and moderate disability) or “unfavorable” (severe disability, persistent vegetative state, and death). Outcomes of all patients, including patients with acute SDH and younger adults (≤50 years of age) with acute SDH, were compared between the hypothermia and fever control groups. The association between head CT findings and clinical demographics and outcomes was also examined in these groups.

### Statistical analysis

Baseline characteristics were summarized as numbers (%) for categorical variables and compared using the chi-square test. Continuous variables were summarized using the median and interquartile ranges and compared using the Mann-Whitney U test. Stepwise multi-variable logistic regression models were built to analyze the independent association variables and outcome measures. Clinical variables with *p* < 0.25 in univariate analysis were selected, excluding those including confounding factors. Entry and exit criteria for the model were 0.25 and 0.1, respectively. All tests were two-sided, with a *p* value of <0.05 indicating statistical significance. All analyses were performed using JMP software (version 14.0.0; SAS Institute Inc., Cary, NC).

## Results

### Outcomes

**FIG. 1.**
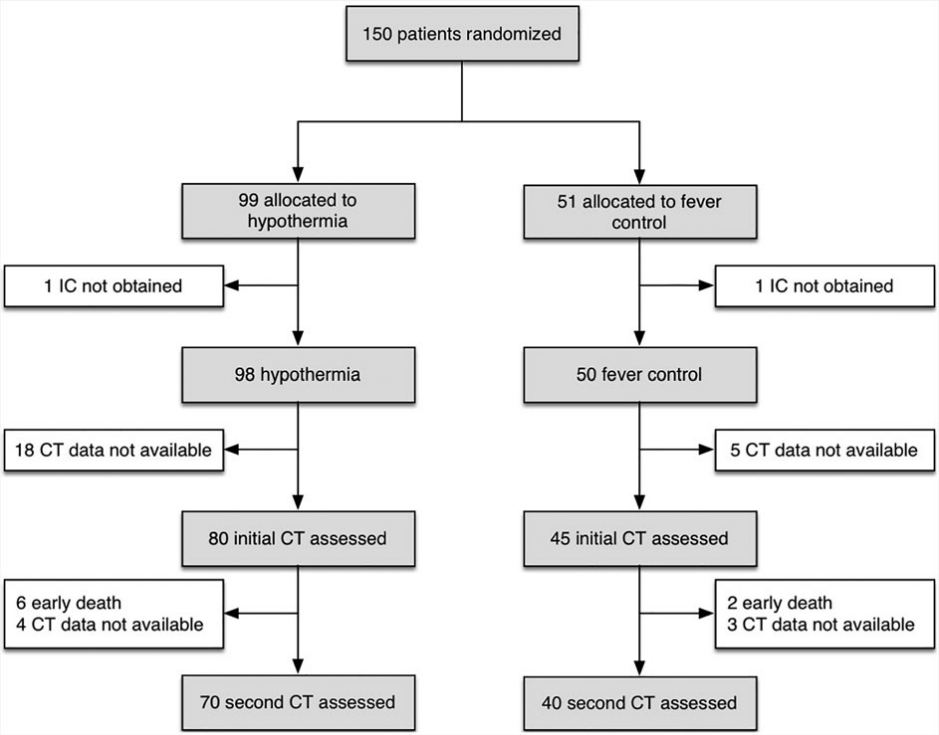
Flowchart of enrolled patients. Among the 150 patients, head CT images taken on arrival were assessed for 80 and 45 patients in the hypothermia and fever control groups, respectively. Head CT images after rewarming (second CT) were assessed in 70 and 40 patients for the hypothermia and fever control groups, respectively. IC, informed consent; CT, computed tomography.

Among 150 randomized patients, initial CT data were evaluated in 125 patients: 80 in the hypothermia group and 45 in the fever control group. Unfortunately, 8 patients died of destructive brain damage before the second CT. Seven patients survived, but the CT data could not be obtained because of privacy policies in some hospitals. Thus, the second CT data were accessible for 110 patients: 70 and 40 in the hypothermia and fever control groups, respectively (Fig. 1).

**FIG. 2.**
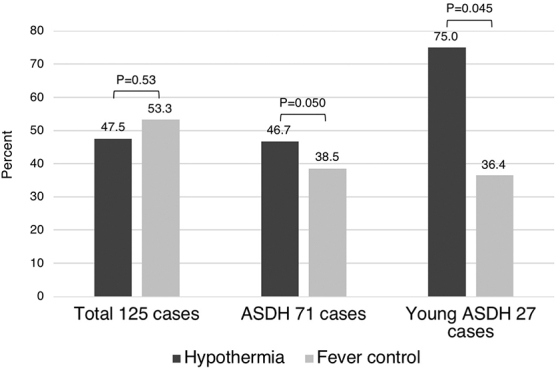
Comparison of favorable outcomes in the hypothermia (32°C–34°C) group and fever control (35.5°C–37°C) groups among the total of 125 cases, 71 ASDH cases, and 27 ASDH cases in younger patients. Treatment with hypothermia significantly improved favorable outcomes (*p* = 0.045) in younger patients with ASDH. ASDH, acute subdural hematoma.

Hypothermia did not improve neurological outcomes or mortality in 125 patients (total) and in 71 patients with acute SDH. Of all patients, 38 (47.5%) in the hypothermia group and 24 (53.3%) in the fever control group had favorable outcomes (*p* = 0.53), with a mortality rate of 27 (33.8%) and 10 (22.2%; *p* = 0.18), respectively. Of the 71 patients with acute SDH, 45 were treated with hypothermia and 26 with fever control. Favorable outcomes were observed in 21 patients (46.7%) in the hypothermia group and 10 patients (38.5%) in the fever control group (*p* = 0.50), with a mortality rate of 18 (40.0%) and 8 (30.8%; *p* = 0.43), respectively. Conversely, in 27 young adults (≤50 years) with acute SDH, hypothermia significantly improved favorable outcomes in 12 of 16 (75.0%) patients with hypothermia and 4 of 11 patients (36.4%) with fever control (*p* = 0.045; Fig. 2). There was no significant difference in mortality, with 2 (12.5%) and 3 (27.3%) patients in the hypothermia and fever control groups, respectively (*p* = 0.33).

### Computed tomography findings of 125 total cases

Table 1 shows baseline characteristics of the 125 patients eligible for head CT assessment. There was no difference in any of the listed variables between the hypothermia and fever control groups. In the hypothermia group, median times to start cooling and reach 35.5°C and then 34.0°C were 160, 280, and 493 min, respectively.

**Table 1.** Baseline Characteristics of the Study Patients (Total 125 Patients)

	Hypothermia	Fever control	
Variable	*n* = 80	*n* = 45	*p* value
Age, years	41 (21–56)	33 (21.5–-56.5)	0.79
Sex, male, *n* (%)	55 (68.8)	31 (68.9)	0.99
Pupil reactivity, *n* (%)			0.82
Both	42 (53.2)	25 (55.6)	
One	10 (12.7)	4 (8.9)	
None	27 (34.2)	16 (35.6)	
GCS motor score	4 (2–4)	4 (2–4)	0.87
ICP, mm Hg	14 (10.0–21.5)	18 (11.5–27.0)	0.13
CPP, mm Hg	69 (57.5–81.5)	69 (56–82)	1.00
IMPACT core	6 (4–8)	6 (3–10)	0.89
IMPACT extended	8 (6–12)	8 (5.0–12.5)	0.80
IMPACT lab	12 (9–15)	10 (8–15)	0.44
Time to cooling initiation, min	160 (120–255)	NA	
Time to 35.5°C, min	280 (210–450)	NA	
Time to 34.0°C, min	493 (307–705)	NA	
Time from injury to surgery, min	180 (145–244)	195 (137.5–244.0)	0.89
Time from arrival to surgery, min	123 (84–195)	130 (106–202)	0.65
Evacuation, *n* (%)	41 (51.3)	22 (48.9)	0.80
Decompressive craniectomy, *n* (%)	38 (47.5)	21 (46.7)	0.93
Bilateral operations, *n* (%)	5 (6.3)	3 (6.7)	0.93

Values are presented as number (%) or median (interquartile range), unless otherwise indicated.

GCS, Glasgow Coma Scale; ICP, intracranial pressure; CPP, cerebral perfusion pressure; IMPACT, International Mission for Prognosis and Analysis of Clinical Trials; NA, not applicable.

Table 2 lists the factors associated with outcomes. Hypothermia was not shown to affect outcome; instead, favorable outcomes and survival rates were related to younger age, lower ICP, higher cerebral perfusion pressure (CPP), and lower IMPACT scores. Evacuation of hematomas and decompressive craniectomies showed a trend toward unfavorable outcomes. Lower GCS motor scores and bilateral operations were associated with unfavorable outcomes.

**Table 2.** Factors Associated with Outcome (Total 125 Patients)

	Favorable	Unfavorable		Alive	Dead	
Variable	*n* = 62	*n* = 63	*p* value	*n* = 88	*n* = 37	*p* value
Age, years	27 (19–48)	53 (30–61)	**<0.0001**	31.5 (19.25–55.00)	53 (28–61)	**0.011**
Sex, male, *n* (%)	46 (74.2)	40 (63.5)	0.20	60 (68.2)	26 (70.3)	0.82
Pupil reactivity, *n* (%)			0.91			0.99
Both	34 (55.7)	33 (52.4)		47 (54.0)	20 (54.1)	
One	7 (11.5)	7 (11.1)		10 (11.5)	4 (10.8)	
None	20 (32.8)	23 (36.5)		30 (34.5)	13 (35.1)	
GCS motor score	4 (3–4)	3 (2–4)	**0.007**	4 (2.25–4.00)	3 (2–4)	0.056
ICP, mm Hg	14 (10.5–20.5)	18 (10.5–38.0)	**0.032**	13 (10–20)	27 (14.5–61.5)	**<0.0001**
CPP, mm Hg	74 (58.5–83.0)	63 (43–77)	**0.033**	75 (60–83)	56 (13.5–65.0)	**<0.0001**
IMPACT core	4 (2–8)	7 (4–10)	**0.0006**	5 (3–8)	7 (5–10)	**0.026**
IMPACT extended	10 (7.25–12.00)	12 (9–12)	**0.0002**	7.5 (5–12)	11 (7.5–13.0)	**0.0065**
IMPACT lab	10 (7.00–12.25)	14 (10–16)	**<0.0001**	10 (8–14)	15 (11–16)	**0.0016**
Hypothermia, *n* (%)	38 (61.3)	42 (66.7)	0.53	53 (60.2)	27 (73.0)	0.18
Time to cooling initiation, min	160 (130–248)	165 (120.0–262.5)	0.99	162.5 (137.50–249.75)	162 (103.0–277.5)	0.72
Time to 35.5°C, min	295 (210.00–456.25)	280 (190.0–442.5)	0.93	297.5 (210.00–453.75)	279 (180–420)	0.71
Time to 34.0°C, min	480 (295–710)	502.5 (340–705)	0.77	510.5 (357.5–742.5)	480 (298–630)	0.36
Time from injury to surgery, min	175 (130.0–277.5)	195 (146.5–246.0)	0.61	200 (141.50–272.25)	180 (140–220)	0.32
Time from arrival to surgery, min	110 (80–210)	130 (100–181)	0.70	149 (83.75–200.50)	121.5 (94.0–169.5)	0.73
Evacuation, *n* (%)	26 (41.9)	37 (58.7)	0.060	42 (47.7)	21 (56.8)	0.36
Decompressive craniectomy, *n* (%)	24 (38.7)	35 (55.6)	0.059	40 (45.5)	19 (51.4)	0.55
Bilateral operations, *n* (%)	1 (1.6)	7 (11.1)	**0.030**	6 (6.8)	2 (5.4)	0.77

Values are presented as number (%) or median (interquartile range), unless otherwise indicated. Boldface type indicates statistical significance.

GCS, Glasgow Coma Scale; ICP, intracranial pressure; CPP, cerebral perfusion pressure; IMPACT, International Mission for Prognosis and Analysis of Clinical Trials.

There were no significant differences in all aspects of CT findings between the two groups (Supplementary Table S2). Table 3 presents the initial CT findings and outcomes. The presence of tSAH, midline shift in millimeters, shift > hematoma thickness, magnitude of basal cistern compression, and a higher Rotterdam CT score as a continuous variable and stratified were associated with unfavorable outcomes. Regarding mortality, the presence of SDH, midline shift in millimeters, shift > hematoma thickness, magnitude of basal cistern compression, and a higher Rotterdam CT score as a continuous variable were all significant.

**Table 3.** Initial Computed Tomographic Findings in Relation to Outcome (Total 125 Patients)

	Favorable	Unfavorable		Alive	Dead	
Variable	*n* = 62	*n* = 63	*p* value	*n* = 88	*n* = 37	*p* value
Laterality, right, *n* (%)	22 (55.0)	26 (54.2)	0.49	32 (54.2)	16 (55.2)	0.46
Bilateral lesions, *n* (%)	5 (8.1)	6 (9.5)	0.77	8 (9.1)	3 (8.1)	0.86
Contusion, *n* (%)	19 (30.7)	23 (36.5)	0.49	30 (34.1)	12 (34.3)	0.86
tSAH, *n* (%)	33 (53.2)	51 (81.0)	**0.0010**	55 (62.5)	29 (78.4)	0.084
EDH, *n* (%)	3 (4.8)	1 (1.6)	0.30	4 (4.6)	0 (0)	0.19
SDH, *n* (%)	31 (50.0)	40 (63.5)	0.13	45 (51.1)	26 (70.3)	**0.049**
Thickness, mm, *n* (%)	14 (8–17)	13 (9.25–17.00)	0.65	15 (9.5–17.5)	12 (8–17)	0.62
<5	6 (19.4)	2 (5.0)	0.11	7 (15.6)	1 (3.9)	0.099
≥5, <10	4 (12.9)	10 (25.0)		6 (13.3)	8 (30.8)	
≥10	21 (67.7)	28 (70.0)		32 (71.1)	17 (65.4)	
Midline shift, mm, *n* (%)	0 (0.0–12.5)	7.5 (0.00–15.75)	**0.025**	0.5 (0.00–13.25)	10 (0–18)	**0.023**
<5	35 (57.4)	22 (36.7)	0.053	46 (53.5)	11 (31.4)	0.082
≥5, <10	7 (11.5)	7 (11.7)		10 (11.6)	4 (11.4)	
≥10, <15	1 (1.6)	6 (10.0)		3 (3.5)	4 (11.4)	
≥15	18 (29.5)	25 (41.7)		27 (31.4)	16 (45.7)	
Shift > thickness, *n* (%)	3 (10.3)	16 (41.0)	**0.0053**	7 (16.3)	12 (48.0)	**0.0049**
Basal cistern, *n* (%)			**0.014**			**0.022**
Normal, *n* (%)	25 (40.3)	15 (23.8)		32 (36.4)	8 (21.6)	
Compressed, *n* (%)	25 (40.3)	21 (33.3)		35 (39.8)	11 (29.7)	
Absent, *n* (%)	12 (19.4)	27 (42.9)		21 (23.9)	18 (48.7)	
Rotterdam Sum Score, *n* (%)	4 (3–4)	5 (4–6)	**0.0002**	4 (3–5)	5 (4–6)	**0.0057**
2	8 (12.9)	2 (3.2)	**0.0013**	9 (10.2)	1 (2.7)	0.058
3	18 (29.0)	13 (20.6)		24 (27.3)	7 (18.9)	
4	24 (38.7)	14 (22.2)		29 (33.0)	9 (24.3)	
5	7 (11.3)	16 (25.4)		15 (17.1)	8 (21.6)	
6	5 (8.1)	18 (28.6)		11 (12.5)	12 (32.4)	
TCDB classification, *n* (%)			0.078			0.059
1	2 (3.2)	0 (0)		2 (2.3)	0 (0)	
2	23 (37.1)	14 (22.2)		31 (35.2)	6 (16.2)	
3	11 (17.7)	10 (15.9)		14 (15.9)	7 (18.9)	
4	0 (0)	3 (4.8)		2 (2.3)	1 (2.7)	
5	25 (40.3)	32 (50.8)		38 (43.2)	19 (51.4)	
6	1 (1.6)	4 (6.4)		1 (1.1)	4 (10.8)	

Values are presented as number (%) or median (interquartile range), unless otherwise indicated. Boldface type indicates statistical significance.

tSAH, traumatic subarachnoid hemorrhage; EDH, epidural hematoma; SDH, subdural hematoma; TCDB, Traumatic Coma Data Bank.

The second CT data were available for 110 patients. In the fever control group, the second CT was often performed earlier, and SDHs with greater hematoma thickness were more common (Supplementary Table S3). The second CT scan was performed earlier in non-surviving patients. Unfavorable outcomes and mortality were related to a left-sided lesion, bilateral lesion, tSAH, stratified midline shift, the magnitude of basal cistern compression, and the Rotterdam CT score as a continuous variable and stratified, respectively. There was a trend toward unfavorable outcomes when the second CT was performed earlier and when the shift > thickness. Intracerebral bleeding related to ICP sensor insertion was observed in 7 patients (6.4%), but did not affect the outcome (Table 4).

**Table 4.** Second Computed Tomographic Findings in Relation to Outcome (Total 110 Patients)

	Favorable	Unfavorable		Alive	Dead	
Variable	*n* = 58	*n* = 52	*p* value	*n* = 82	*n* = 28	*p* value
Second CT, day	7 (7.00–7.25)	7 (6–7)	0.074	7 (7.0–7.5)	7 (2–7)	**0.0007**
Laterality, right, *n* (%)	20 (43.5)	14 (31.8)	**0.024**	29 (44.6)	5 (20.0)	**0.038**
Bilateral lesions, *n* (%)	8 (13.8)	30 (57.7)	**<0.0001**	19 (23.2)	19 (67.9)	**<0.0001**
Contusion, *n* (%)	37 (63.8)	38 (73.1)	0.30	53 (64.6)	22 (78.6)	0.17
tSAH, *n* (%)	4 (6.9)	15 (28.9)	**0.0024**	9 (11.0)	10 (35.7)	**0.0028**
EDH, *n* (%)	5 (8.6)	2 (3.9)	0.31	5 (6.1)	2 (7.1)	0.84
SDH, *n* (%)	7 (12.1)	4 (7.7)	0.44	9 (11.0)	2 (7.1)	0.56
Thickness, mm, *n* (%)	0 (0–0)	0 (0–0)	0.48	0 (0–0)	0 (0–0)	0.58
<5	51 (87.9)	48 (92.3)	0.31	73 (89.0)	26 (92.9)	0.77
≥5, <10	7 (12.1)	3 (5.8)		8 (9.8)	2 (7.1)	
≥10	0 (0)	1 (1.9)		1 (1.2)	0 (0)	
Midline shift, mm, *n* (%)	0 (0.00–0.25)	0 (0.00–3.75)	**0.0094**	0 (0–2)	0 (0.0–4.5)	0.19
<5	44 (75.9)	29 (55.8)	**0.039**	56 (68.3)	17 (60.7)	**0.033**
≥5, <10	11 (19.0)	15 (28.9)		21 (25.6)	5 (17.9)	
≥10, <15	3 (5.2)	3 (5.8)		4 (4.9)	2 (7.1)	
≥15	0 (0)	5 (9.6)		1 (1.2)	4 (14.3)	
Shift > thickness, *n* (%)	12 (20.7)	19 (37.3)	0.056	20 (24.7)	11 (39.3)	0.14
Basal cistern, n (%)			**<0.0001**			**<0.0001**
Normal	54 (93.1)	30 (57.7)		71 (86.6)	13 (46.4)	
Compressed	4 (6.9)	9 (17.3)		11 (13.4)	2 (7.1)	
Absent	0 (0)	13 (25.0)		0 (0)	13 (46.4)	
Rotterdam Sum Score, *n* (%)	2 (2–3)	3 (2–5)	**<0.0001**	2 (2–3)	3.5 (2.25–5.00)	**<0.0001**
1	1 (1.7)	0 (0)	**0.0003**	1 (1.2)	0 (0)	**<0.0001**
2	35 (60.3)	16 (30.8)		44 (53.7)	7 (25.0)	
3	19 (32.8)	16 (30.8)		28 (34.2)	7 (25.0)	
4	3 (5.2)	6 (11.5)		8 (9.8)	1 (3.6)	
5	0 (0)	10 (19.2)		1 (1.2)	9 (32.1)	
6	0 (0)	4 (7.7)		0 (0)	4 (14.3)	
ICP bleeding, *n* (%)	3 (5.2)	4 (7.7)	0.59	4 (4.9)	3 (10.7)	0.27

Values are presented as number (%) or median (interquartile range), unless otherwise indicated. Boldface type indicates statistical significance.

tSAH, traumatic subarachnoid hemorrhage; EDH, epidural hematoma; SDH, subdural hematoma; ICP, intracranial pressure.

### Computed tomography findings of 27 young adults with acute subdural hematoma

Baseline characteristics (Supplementary Table S4) and initial CT findings (Supplementary Table S5) of the 27 young adults with acute SDH were similar in all aspects of the hypothermia and fever control groups. Hypothermia was related to favorable outcomes, and there was a trend toward favorable outcomes for rapidly reaching 35.5°C (190 vs. 377 min, *p* = 0.052) and for surgery (155 vs. 180 min, *p* = 0.096). Pupil reactivity, ICP, CPP, and IMPACT scores were not associated with favorable outcomes. For mortality, GCS motor scores and IMPACT scores were significant, but were not relevant to favorable outcomes (Table 5).

**Table 5.** Factors Associated with Outcome (27 Young Patients with Acute Subdural Hematoma)

	Favorable	Unfavorable		Alive	Dead	
Variable	*n* = 16	*n* = 11	*p* value	*n* = 22	*n* = 5	*p* value
Age, years (95% CI)	23 (18.25–41.50)	32 (21–42)	0.22	27 (19.75–43.75)	21 (20.5–34.5)	0.55
Sex, male, *n* (%)	10 (62.5)	8 (72.7)	0.58	14 (63.6)	4 (80)	0.48
Pupil reactivity, *n* (%)			0.59			0.32
Both	4 (25.0)	3 (27.3)		7 (31.8)	0 (0)	
One	1 (6.3)	2 (18.8)		2 (9.1)	1 (20.0)	
None	11 (68.8)	6 (54.6)		13 (59.1)	4 (80.0)	
GCS motor score	3.5 (2–4)	3 (2–4)	0.19	3.5 (2–4)	2 (1.5–2.5)	**0.021**
ICP, mm Hg	15 (12.25–17.75)	18 (12–54)	0.23	15 (12.00–19.25)	54 (14–101)	0.074
CPP, mm Hg	77 (56.75–82.50)	60 (22–83)	0.31	75.5 (58.25–83.00)	22 (9.5–79.5)	0.086
IMPACT core	8 (4.0–9.5)	8 (6–10)	0.44	7.5 (4–9)	10 (8–11)	**0.050**
IMPACT extended	10 (6.5–12.0)	12 (9–12)	0.30	10 (6.75–12.00)	12 (12–13)	**0.027**
IMPACT lab	12 (9.25–15.75)	15 (12–16)	0.17	12 (9.75–15.00)	16 (16–17)	**0.0069**
Hypothermia, *n* (%)	12 (75.0)	4 (36.4)	**0.045**	14 (63.6)	2 (40.0)	0.33
Time to 35.5°C, min	190 (135.00–296.25)	377 (256.0–1162.5)	0.052	235 (145.00–336.25)	325 (230–420)	0.43
Time to 34.0°C, min	297.5 (247.50–554.25)	495 (393–1410)	0.18	330 (252.5–715.0)	495 (480–510)	0.53
Time from arrival to surgery, min	93 (76–162)	128 (108–308)	0.15	98 (78.0–192.5)	120 (94.5–143.5)	0.92
Time from injury to surgery, min	155 (135–197)	180 (165–334)	0.096	165 (141–234)	165 (130.5–190.0)	0.56
Decompressive craniectomy, *n* (%)	14 (87.5)	10 (90.9)	0.78	20 (90.9)	4 (80.0)	0.48
Bilateral operations, *n* (%)	1 (6.3)	2 (18.2)	0.33	3 (13.6)	0 (0)	0.38
						

Values are presented as number (%) or median (interquartile range), unless otherwise indicated. Boldface type indicates statistical significance.

CI, confidence interval; GCS, Glasgow Coma Scale; ICP, intracranial pressure; CPP, cerebral perfusion pressure; IMPACT, International Mission for Prognosis and Analysis of Clinical Trials.

We assessed the association between independent variables and outcomes based on multi-variable logistic regression models. All variables with a *p* value <0.25 in Table 5 were included. Regarding favorable outcomes, ICP was significant (*p* = 0.047), whereas hypothermia was marginally significant (*p* = 0.059), and time from injury to surgery (*p* = 0.087), GCS motor score (*p* = 0.23), and age (*p* = 0.34) did not reach significance. For mortality, ICP (*p* = 0.0013) and GCS motor score (*p* = 0.017) were significant (Supplementary Table S6).

A trend toward unfavorable outcomes was observed in patients with tSAH. The Rotterdam CT score as a continuous variable was significantly associated with unfavorable outcomes. No prognostic factors were identified for mortality (Table 6).

**Table 6.** Initial Computed Tomographic Findings in Relation to Outcome (27 Young Patients with Acute Subdural Hematoma)

	Favorable	Unfavorable		Alive	Dead	
Variable	*n* = 16	*n* = 11	*p* value	*n* = 22	*n* = 5	*p* value
Laterality, right, *n* (%)	10 (62.5)	6 (54.6)	0.68	13 (59.1)	3 (60.0)	0.97
Bilateral lesions, *n* (%)	0 (0)	0 (0)		0 (0)	0 (0)	
Contusion, *n* (%)	4 (25.0)	4 (36.4)	0.53	7 (31.8)	1 (20.0)	0.60
tSAH, *n* (%)	6 (37.5)	8 (72.7)	0.072	12 (54.5)	2 (40.0)	0.56
EDH, *n* (%)	0 (0)	0 (0)		0 (0)	0 (0)	
SDH, *n* (%)	16 (100)	11 (100)		22 (100)	5 (100)	
Thickness, mm, *n* (%)	14.5 (10.25–19.50)	15 (11–17)	0.98	15 (9.75–19.00)	15 (11.5–15.0)	0.80
<5	2 (12.5)	1 (9.1)	0.92	3 (13.6)	0 (0)	0.42
≥5, <10	2 (12.5)	1 (9.1)		3 (13.6)	0 (0)	
≥10	12 (75.0)	9 (81.8)		16 (72.7)	5 (100)	
Midline shift, mm, *n* (%)	12.5 (6.25–15.00)	14 (7–18)	0.66	12.5 (5–15)	15 (8.5–20.0)	0.29
<5	3 (18.8)	0 (0)	0.29	3 (13.6)	0 (0)	0.11
≥5, <10	1 (6.3)	2 (18.2)		3 (13.6)	0 (0)	
≥10, <15	1 (6.3)	2 (18.2)		1 (4.6)	2 (40.0)	
≥15	11 (68.8)	7 (63.6)		15 (68.2)	3 (60.0)	
Shift > thickness, *n* (%)	2 (12.5)	3 (27.3)	0.33	3 (13.6)	2 (40.0)	0.17
Basal cistern, *n* (%)			0.29			0.46
Normal	1 (6.3)	0 (0)		1 (4.6)	0 (0)	
Compressed	8 (50.0)	3 (27.3)		10 (90.9)	1 (20.0)	
Absent	7 (43.8)	8 (72.7)		11 (50.0)	4 (80.0)	
Rotterdam Sum Score, *n* (%)	4.5 (4–5)	5 (5–6)	**0.025**	5 (4–5)	5 (4.5–6.0)	0.30
2	0 (0)	0 (0)	0.14	0 (0)	0 (0)	0.72
3	1 (6.3)	0 (0)		1 (4.6)	0 (0)	
4	7 (43.8)	1 (20.0)		7 (31.8)	1 (20.0)	
5	6 (37.5)	6 (54.6)		10 (45.5)	2 (40.0)	
6	2 (12.5)	4 (36.4)		4 (18.2)	2 (40.0)	

Values are presented as number (%) or median (interquartile range), unless otherwise indicated. Boldface type indicates statistical significance.

tSAH, traumatic subarachnoid hemorrhage; EDH, epidural hematoma; SDH, subdural hematoma.

All patients survived until the second CT scan, which showed that bilateral lesions developed more in the fever control group, while other parameters were similar between the two groups (Supplementary Table S7). Bilateral lesions, tSAH, midline shift in mm, and Rotterdam CT score as a continuous variable were significantly associated with unfavorable outcomes. A significant correlation for mortality was observed in the midline shift in millimeters and stratified, the magnitude of the basal cistern compression, and Rotterdam CT score as a continuous variable and stratified. A trend toward higher mortality was observed in patients with bilateral lesions (Table S8).

## Discussion

There was no difference in detailed head CT findings between the hypothermia and fever control groups. Early induction of hypothermia significantly increased favorable outcomes in patients with acute SDH ≤50 years of age, from 36.4% in the fever control group to 75.0% in the hypothermia group. Less time to reach 35.5°C and earlier surgical intervention were associated with a favorable outcome. However, these findings may not be conclusive. Multi-variate analysis showed that the benefits of hypothermia were marginal, and early surgery was associated with a trend toward favorable outcomes. Conversely, a trend toward worse outcomes was evident in the subgroups with hematoma evacuation and decompressive craniectomy for all 125 patients. These findings imply that acute SDH does not necessarily justify hypothermia treatment. However, young adults with acute SDH may benefit more from the rapid induction of hypothermia combined with surgical intervention.

We previously reported that favorable outcomes increased significantly with hypothermia treatment in young adults (≤50 years) with evacuated mass lesions.^15^ The present study confirmed the benefits of hypothermia in young adults with acute SDH, excluding intraparenchymal hematoma. We also confirmed the association between the time course and surgery. More favorable outcomes were noted in patients in whom cooling and surgery were started almost simultaneously (147.5 and 155 min after injury, respectively), and body temperature reached 35.5°C 35 min after surgery commenced. These findings correspond with those of a meta-analysis conducted by the National Acute Brain Injury Study: Hypothermia (NABIS:H) I and II, which showed that lowering body temperature to 35°C before or soon after craniotomy improved the outcomes of patients with severe TBI and hematomas.^14^

The Prophylactic Hypothermia Trial to Lessen Traumatic Brain Injury–Randomized Clinical Trial (POLAR-RCT) showed that hypothermia did not improve neurological outcomes, either for the whole study group or in the subgroups with surgically removed hematomas or any intracranial mass lesions.^8^ No details of subgroup analyses were provided. These results were unsurprising, given that the presence of mass lesions in patients >45 years of age tends to yield more unfavorable outcomes.^19^ Our study also demonstrated a trend toward worse outcomes in the subgroups with hematoma evacuation.

TBI is a heterogeneous, highly complex clinical condition, and the location and nature of the brain damage can vary among patients. The Rotterdam CT score,^18^ presence of tSAH,^20^ and magnitude of midline shift as a continuous variable^21,22^ have all been reported as significant prognosticators in TBI. Our study determined that clinical baseline characteristics and initial CT parameters were useful in predicting outcomes of all patients treated with TTM. Interestingly, in young adults with acute SDH, prognostic factors, such as ICP, CCP, and IMPACT scores, were not associated with functional outcomes, but only with death. On the initial CT scan, only a higher Rotterdam CT score as a continuous variable predicted an unfavorable outcome.

The second CT findings may reflect progression of the brain injury. In all patients, incidence of SDH and its thickness were significantly greater in the fever control group. Massive injury in the dominant hemisphere may be associated with poor functional outcomes and medical futility. In young patients with acute SDH, bilateral lesions developed more frequently in the fever control group and were associated with unfavorable outcomes. Hypothermia may be attributable to fewer bilateral injuries. This finding suggests that hypothermia attenuated brain damage not only on the acute SDH side, but also on the opposite side.

A cascade of destructive events and processes begins at the cellular level in the minutes and hours after the initial injury.^23^ In a meta-analysis of hypothermia in experimental TBI, cooling was initiated early: The delay was mostly within 1 h post-injury, with greater efficacy with earlier treatment.^10^ Delayed hypothermia induction is hypothesized as the primary reason why hypothermia is not beneficial in clinical practice.

Thus far, the benefits of hypothermia treatment have been clinically shown in whole-brain ischemia/reperfusion injury in adult post-cardiac arrest^24,25^ and neonatal hypoxic-ischemic encephalopathy.^26^ In an acute SDH rat model, ipsilateral focal ischemia and edema developed after hematoma removal, causing significant hemispheric swelling.^27^ Early, pre-operatively induced hypothermia reduced neuronal degeneration and injury volume by attenuating neuronal and glial cell damage in this particular ischemia/reperfusion model.^28^ Although the mechanism underlying the consequences of TBI is complicated, focal post-ischemic reperfusion injury is postulated to be crucial in the pathogenesis of brain injury in patients with acute SDH. Therefore, hypothermia is likely to be effective in treating acute SDH attributable to unilateral post-ischemic reperfusion injury. Our results are consistent with these experimental findings. Further, decreased bilateral injury suggests potential benefits beyond mitigating ipsilateral ischemia/reperfusion injury beneath the SDH.

An intravascular cooling system may be useful given that rapid induction, accurate maintenance, and scheduled rewarming can perform easily.^29^ To study the effects of early hypothermia in acute SDH, the Hypothermia for Patients requiring Evacuation of Subdural Hematoma (HOPES) trial—an international RCT using intravascular cooling for rapid cooling with surgery—was conducted.^30^ Unfortunately, the trial was halted because of slow recruitment and medical futility. At the interim futility analysis of 32 patients, there was no difference identified between the hypothermia and normothermia groups in functional outcome. Nevertheless, our study and subanalysis of NABIS:H studies “HOPES” for hypothermia in younger patients with a severe TBI and acute SDH.

### Limitations

There are some limitations to this study. First, this was a retrospective *post hoc* analysis of the B-HYPO study. The original RCT aimed to enroll 300 patients; however, only 150 patients were enrolled, so that the statistical power may be insufficient. Second, CT data were not obtained from all patients, and the quality varied depending on the facility. Third, because this was a CT-based study, the combined DAI could not be completely distinguished. Finally, advanced cooling systems, such as gel pads or intravascular cooling devices with automated temperature control, are unavailable.

## Conclusion

Regarding CT findings, there was no difference in the patients' allocation in the B-HYPO study. However, under similar CT findings, early induction of hypothermia combined with early surgical evacuation increased favorable outcomes in young adults with acute SDH compared to those undergoing fever control. In addition, there was a trend toward favorable outcomes for patients who took less time to reach 35.5°C and for earlier surgical intervention.

## Supplementary Material

Supplemental data

Supplemental data

Supplemental data

Supplemental data

Supplemental data

Supplemental data

Supplemental data

Supplemental data

## Abbreviations Used

B-HYPO: brain hypothermia
CPP: cerebral perfusion pressure
CT: computed tomography
DAI: diffuse axonal injury
EDH: epidural hematoma
GCS: Glasgow Coma Scale
ICP: intracranial pressure
IMPACT: International Mission for Prognosis and Analysis of Clinical Trials
IVH: intraventricular hematoma
RCT: randomized controlled trial
SDH: subdural hematoma
TBI: traumatic brain injury
TCDB: Traumatic Coma Data Bank
tSAH: traumatic subarachnoid hemorrhage
TTM: targeted temperature management
UMIN: University Hospital Medical Information Network
