# Supplementary material for: Benefits of Hypothermia for Young Patients with Acute Subdural Hematoma: A Computed Tomography Analysis of the Brain Hypothermia Study
Source: Neurotrauma Rep. 2022 Jul 15;3(1):250–60. doi: 10.1089/neur.2021.0080 (PMC9380885; doi:10.1089/neur.2021.0080)
Supplement: Supplemental data [file Supp_TableS1.docx]

Supplementary Table S1. The Rotterdam computed tomographic score

| Predictor values | Score |
| --- | --- |
| Basal cisterns |  |
| Normal | 0 |
| Compressed | 1 |
| Absent | 2 |
| Midline shift |  |
| No shift or shift ≤5 mm | 1 |
| Shift >5 mm | 2 |
| Epidural mass lesion |  |
| Present | 0 |
| Absent | 1 |
| Intraventricular blood or tSAH |  |
| Absent | 0 |
| Present | 1 |
| Sum score | +1 |

tSAH, traumatic subarachnoid hemorrhage.

One point is added the sum score to make the Rotterdam grade numerically total 6 points, based on Maas et al. ^18^
