# Supplementary material for: Benefits of Hypothermia for Young Patients with Acute Subdural Hematoma: A Computed Tomography Analysis of the Brain Hypothermia Study
Source: Neurotrauma Rep. 2022 Jul 15;3(1):250–60. doi: 10.1089/neur.2021.0080 (PMC9380885; doi:10.1089/neur.2021.0080)
Supplement: Supplemental data [file Supp_TableS2.docx]

Supplementary Table S2. Initial computed tomographic findings in relation to target temperature (total 125 patients)

| Variable | Hypothermia | Fever control | p value |
| --- | --- | --- | --- |
|  | n= 80 | n = 45 |  |
| Laterality, right, n (%) | 32 (56.1) | 16 (51.6) | 0.68 |
| Bilateral lesions, n (%) | 8 (10) | 3 (6.7) | 0.53 |
| Contusion, n (%) | 27 (33.8) | 15 (33.3) | 0.96 |
| SAH, n (%) | 56 (70.0) | 28 (62.2) | 0.37 |
| EDH, n (%) | 2 (2.5) | 2 (4.4) | 0.55 |
| SDH, n (%) | 45 (56.3) | 26 (57.8) | 0.87 |
| Thickness, mm | 14 (9.5–17) | 13 (8–17) | 0.50 |
| <5 mm, n (%) | 4 (15.4) | 4 (8.9) | 0.55 |
| ≥5, <10 mm, n (%) | 8 (23.1) | 6 (17.8) |  |
| ≥10 mm, n (%) | 33 (61.5) | 16 (73.3) |  |
| Midline shift, n | 3 (0–15) | 2 (0–12) | 0.57 |
| <5 mm, n (%) | 36 (46.2) | 21 (48.9) | 0.57 |
| ≥5, <10 mm, n (%) | 9 (11.5) | 5 (11.6) |  |
| ≥10, <15 mm, n (%) | 3 (3.9) | 4 (9.3) |  |
| ≥15 mm, n (%) | 30 (38.5) | 13 30.2) |  |
| Shift > thickness, n (%) | 15 (34.1) | 4 (16.7) | 0.13 |
| Basal cistern, n (%) |  |  | 0.97 |
| Normal, n (%) | 25 (31.3) | 15 (31.1) |  |
| Compressed, n (%) | 30 (37.5) | 16 (35.6) |  |
| Absent, n (%) | 25 (31.3) | 14 (31.1) |  |
| Rotterdam Sum Score | 4 (3–5) | 4 (3–5) | 0.69 |
| 2, n (%) | 6 (7.5) | 4 (8.9) | 0.99 |
| 3, n (%) | 19 (23.8) | 12 (26.7) |  |
| 4, n (%) | 25 (31.3) | 13 (28.9) |  |
| 5, n (%) | 15 (17.8) | 8 (17.8) |  |
| 6, n (%) | 15 (18.8) | 8 (17.8) |  |
| TCDB classification |  |  | 0.36 |
| 1, n (%) | 1 (2.2) | 1 (2.2) |  |
| 2, n (%) | 22 (27.5) | 15 (33.3) |  |
| 3, n (%) | 12 (15.0) | 9 (20.0) |  |
| 4, n (%) | 1 (1.3) | 2 (4.4) |  |
| 5, n (%) | 39 (48.8) | 18 (40.0) |  |
| 6, n (%) | 5 (6.3) | 0 (0) |  |

tSAH, traumatic subarachnoid hemorrhage; EDH, epidural hematoma; SDH, subdural hematoma; TCDB, traumatic coma data bank.

Values are presented as number (%) or median (interquartile range) unless otherwise indicated.
