# Supplementary material for: Benefits of Hypothermia for Young Patients with Acute Subdural Hematoma: A Computed Tomography Analysis of the Brain Hypothermia Study
Source: Neurotrauma Rep. 2022 Jul 15;3(1):250–60. doi: 10.1089/neur.2021.0080 (PMC9380885; doi:10.1089/neur.2021.0080)
Supplement: Supplemental data [file Supp_TableS3.docx]

Supplementary Table S3. Second CT findings in relation to target temperature (total 110 patients)

| Variable | Hypothermia | Fever control | p value |
| --- | --- | --- | --- |
|  | n = 70 | n = 40 |  |
| 2nd CT, day | 7 (6–8) | 7 (6–7) | 0.087 |
| Laterality, right, n (%) | 23 (40.4) | 11 (33.3) | 0.77 |
| Bilateral lesions, n (%) | 22 (31.4) | 16 (40.0) | 0.36 |
| Contusion, n (%) | 49 (0.70) | 26 (0.65) | 0.59 |
| tSAH, n (%) | 13 (18.6) | 6 (15.0) | 0.63 |
| EDH, n (%) | 6 (8.6) | 1 (2.5) | 0.21 |
| SDH, n (%) | 4 (5.7) | 7 (17.5) | **0.048** |
| Thickness, mm | 0 (0–0) | 0 (0–0) | **0.039** |
| <5 mm, n (%) | 66 (94.3) | 33 (82.5) | 0.10 |
| ≥5, <10 mm, n (%) | 4 (5.7) | 6 (15.0) |  |
| ≥10 mm, n (%) | 0 (0) | 1 (2.5) |  |
| Midline shift, mm | 0 (0–2) | 0 (0–3) | 0.53 |
| <5 mm, n (%) | 47 (67.1) | 26 (65.0) | 0.90 |
| ≥5, <10 mm, n (%) | 17 (24.3) | 9 (22.5) |  |
| ≥10, <15 mm, n (%) | 3 (4.3) | 3 (7.5) |  |
| ≥15 mm, n (%) | 3 (4.3) | 2 (5.0) |  |
| Shift > thickness, n (%) | 21 (30.0) | 10 (25.6) | 0.63 |
| Basal cistern, n (%) |  |  | 0.71 |
| Normal, n (%) | 55 (78.6) | 29 (72.5) |  |
| Compressed, n (%) | 8 (11.4) | 5 (12.5) |  |
| Absent, n (%) | 7 (10.0) | 6 (15.0) |  |
| Rotterdam Sum Score | 3 (2–3) | 3 (2–3.75) | 0.71 |
| 1, n (%) | 1 (1.4) | 0 (0) | 0.60 |
| 2, n (%) | 32 (45.7) | 19 (47.5) |  |
| 3, n (%) | 24 (34.3) | 11 (27.5) |  |
| 4, n (%) | 6 (8.6) | 3 (7.5) |  |
| 5, n (%) | 4 (5.7) | 6 (15.0) |  |
| 6, n (%) | 3 (4.3) | 1 (2.5) |  |
| ICP bleeding | 5 (7.1) | 2 (5.0) | 0.66 |

CT, computed tomography; tSAH, traumatic subarachnoid hemorrhage; EDH, epidural hematoma; SDH, subdural hematoma; ICP, intracranial pressure.

Values are presented as number (%) or median (interquartile range) unless otherwise indicated. Boldface type indicates statistical significance.
