# Supplementary material for: Benefits of Hypothermia for Young Patients with Acute Subdural Hematoma: A Computed Tomography Analysis of the Brain Hypothermia Study
Source: Neurotrauma Rep. 2022 Jul 15;3(1):250–60. doi: 10.1089/neur.2021.0080 (PMC9380885; doi:10.1089/neur.2021.0080)
Supplement: Supplemental data [file Supp_TableS4.docx]

Supplementary Table S4. Baseline characteristics of the study patients (27 young patients with acute subdural hematoma)

| Variable | Hypothermia | Fever control | p value |
| --- | --- | --- | --- |
|  | n = 16 | n = 11 |  |
| Age, years | 23 (19.25–40.25) | 30 (22–-42) | 0.31 |
| Sex, male, n (%) | 10 (62.5) | 8 (72.7) | 0.58 |
| Pupil reactivity, n (%) |  |  | 0.68 |
| Both | 5 (31.3) | 2 (18.2) |  |
| One | 2 (12.5) | 1 (9.1) |  |
| None | 9 (56.3) | 8 (72.7) |  |
| GCS motor score | 3 (2–4) | 3 (2–4) | 0.86 |
| ICP, mmHg | 15 (12–17.75) | 18 (13-27) | 0.14 |
| CPP, mmHg | 59.5 (60.25–83.75) | 74 (60-81) | 0.82 |
| IMPACT core | 8 (4–9.5) | 8 (6–10) | 0.50 |
| IMPACT extended | 10 (6.5–12) | 12 (8–13) | 0.38 |
| IMPACT lab | 12.5 (9.25–16) | 15 (12–16) | 0.38 |
| Time to cooling initiation, min | 152.5 (105–236.5) | NA |  |
| Time to 35.5°C, min | 242.5 (152.5–340.75) | NA |  |
| Time to 34.0°C, min | 362 (263.75–554.25) | NA |  |
| Time from arrival to surgery, min | 97 (82–162) | 136 (65–235) | 0.45 |
| Time from injury to surgery, min | 155 (135–202) | 180(165–285) | 0.20 |
| Decompressive craniectomy, n (%) | 14 (87.5) | 10 (90.9) | 0.78 |
| Bilateral operations, n (%) | 1 (6.3) | 2 (18.2) | 0.33 |

GCS, Glasgow Coma Scale; ICP, intracranial pressure; CPP, cerebral perfusion pressure; IMPACT, International Mission for Prognosis and Analysis of Clinical Trials; NA, not applicable.

Values are presented as number (%) or median (interquartile range) unless otherwise indicated.
