# Supplementary material for: Benefits of Hypothermia for Young Patients with Acute Subdural Hematoma: A Computed Tomography Analysis of the Brain Hypothermia Study
Source: Neurotrauma Rep. 2022 Jul 15;3(1):250–60. doi: 10.1089/neur.2021.0080 (PMC9380885; doi:10.1089/neur.2021.0080)
Supplement: Supplemental data [file Supp_TableS5.docx]

Supplementary Table S5. Initial computed tomographic findings in relation to target temperature (27 young patients with acute subdural hematoma)

| Variable | Hypothermia | Fever control | p value |
| --- | --- | --- | --- |
|  | n = 16 | n = 11 |  |
| Laterality, right, n (%) | 10 (62.5) | 6 (54.6) | 0.68 |
| Bilateral lesions, n (%) | 0 (0) | 0 (0) |  |
| Contusion, n (%) | 4 (25.0) | 4 (36.4) | 0.53 |
| tSAH, n (%) | 8 (50.0) | 6 (54.6) | 0.82 |
| EDH, n (%) | 0 (0) | 0 (0) |  |
| SDH, n (%) | 16 (100) | 11 (100) | 0.41 |
| Thickness, mm | 15 (11–19.75) | 14 (10–17) | 0.33 |
| <5 mm, n (%) | 1 (6.3) | 2 (18.2) | 0.62 |
| ≥5, <10 mm, n (%) | 2 (12.5) | 1 (9.1) |  |
| ≥10 mm, n (%) | 13 (81.3) | 8 (72.7) |  |
| Midline shift, mm | 13.5 (6.25–15) | 12 (7–16) | 0.98 |
| <5 mm, n (%) | 2 (12.5) | 1 (9.1) | 0.80 |
| ≥5, <10 mm, n (%) | 2 (12.5) | 1 (9.1) |  |
| ≥10, <15 mm, n (%) | 1 (6.3) | 2 (18.2) |  |
| ≥15 mm, n (%) | 11 (68.8) | 7 (63.6) |  |
| Shift > thickness, n (%) | 2 (12.5) | 3 (27.3) | 0.33 |
| Basal cistern, n (%) |  |  | 0.61 |
| Normal, n (%) | 1 (6.3) | 0 (0) |  |
| Compressed, n (%) | 7 (43.8) | 4 (36.4) |  |
| Absent, n (%) | 8 (50.0) | 7 (63.4) |  |
| Rotterdam Sum Score | 5 (4–5) | 5 (4–6) | 0.48 |
| 2, n (%) | 0 (0) | 0 (0) | 0.82 |
| 3, n (%) | 1 (6.3) | 0 (0) |  |
| 4, n (%) | 5 (31.3) | 3 (27.3) |  |
| 5, n (%) | 7 (43.8) | 5 (45.5) |  |
| 6, n (%) | 3 (18.8) | 3 (27.3) |  |

tSAH, traumatic subarachnoid hemorrhage; EDH, epidural hematoma; SDH, subdural hematoma.

Values are presented as number (%) or median (interquartile range) unless otherwise indicated.
