# Supplementary material for: Benefits of Hypothermia for Young Patients with Acute Subdural Hematoma: A Computed Tomography Analysis of the Brain Hypothermia Study
Source: Neurotrauma Rep. 2022 Jul 15;3(1):250–60. doi: 10.1089/neur.2021.0080 (PMC9380885; doi:10.1089/neur.2021.0080)
Supplement: Supplemental data [file Supp_TableS6.docx]

Supplementary Table S6. Results of multiple logistic regression analysis (27 young patients with acute subdural hematoma)

| For favorable outcomes |  |
| --- | --- |
| Variable | p value |
| Intracranial pressure | 0.047 |
| Hypothermia | 0.059 |
| Time from injury to surgery | 0.087 |
| GCS motor score | 0.23 |
| Age | 0.34 |
|  |  |
| For mortality |  |
| Variable | p value |
| Intracranial pressure | 0.0013 |
| GCS motor score | 0.017 |

GCS, Glasgow Coma Scale; ICP, intracranial pressure
