# Supplementary material for: Benefits of Hypothermia for Young Patients with Acute Subdural Hematoma: A Computed Tomography Analysis of the Brain Hypothermia Study
Source: Neurotrauma Rep. 2022 Jul 15;3(1):250–60. doi: 10.1089/neur.2021.0080 (PMC9380885; doi:10.1089/neur.2021.0080)
Supplement: Supplemental data [file Supp_TableS7.docx]

Supplementary Table S7. Second computed tomographic findings in relation to target temperature (27 young patients with acute subdural hematoma)

| Variable | Hypothermia | Fever control | p value |
| --- | --- | --- | --- |
|  | n = 16 | n = 11 |  |
| 2nd CT, day | 7 (6–7.75) | 7 (7–7) | 0.56 |
| Laterality, right, n (%) | 9 (56.3) | 4 (36.4) | 0.16 |
| Bilateral lesions, n (%) | 1 (6.3) | 6 (54.6) | **0.0049** |
| Contusion, n (%) | 12 (75.0) | 7 (63.6) | 0.53 |
| SAH | 3 (18.8) | 2 (18.2) | 0.97 |
| EDH, n (%) | 2 (12.5) | 1 (9.1) | 0.78 |
| SDH, n (%) | 3 (18.8) | 1 (9.1) | 0.49 |
| Thickness, mm | 0 (0–0) | 0 (0–0) | 0.50 |
| <5 mm, n (%) | 13 (81.3) | 10 (90.9) | 0.49 |
| ≥5, <10 mm, n (%) | 3(18.8) | 1 (9.1) |  |
| ≥10 mm, n (%) | 0 (0) | 0 (0) |  |
| Midline shift, mm | 1 (0–2) | 3 (0–8) | 0.24 |
| <5 mm, n (%) | 8 (50.0) | 4 (36.4) | 0.73 |
| ≥5, <10 mm, n (%) | 5 (31.3) | 3 (27.3) |  |
| ≥10, <15 mm, n (%) | 2 (12.5) | 2 (18.2) |  |
| ≥15 mm, n (%) | 1 (6.3) | 2 (18.2) |  |
| Shift > thickness, n (%) | 7 (43.8) | 6 (54.6) | 0.58 |
| Basal cistern, n (%) |  |  | 0.11 |
| Normal, n (%) | 15 (93.8) | 7 (63.4) |  |
| Compressed, n (%) | 0 (0) | 2 (18.2) |  |
| Absent, n (%) | 1 (6.3) | 2 (18.2) |  |
| Rotterdam Sum Score | 2 (2–2.75) | 2 (2–5) | 0.43 |
| 2 | 12 (75.0) | 7 (63.6) | 0.32 |
| 3 | 3 (18.8) | 1 (9.1) |  |
| 4 | 0 (0) | 0 (0) |  |
| 5 | 0 (0) | 2 (18.2) |  |
| 6 | 1 (6.3) | 1 (9.1) |  |
| ICP bleeding | 1 (6.3) | 0 (0) | 0.40 |

tSAH, traumatic subarachnoid hemorrhage; EDH, epidural hematoma; SDH, subdural hematoma; ICP, intracranial pressure.

Values are presented as number (%) or median (interquartile range) unless otherwise indicated. Boldface type indicates statistical significance.
