# Supplementary material for: Benefits of Hypothermia for Young Patients with Acute Subdural Hematoma: A Computed Tomography Analysis of the Brain Hypothermia Study
Source: Neurotrauma Rep. 2022 Jul 15;3(1):250–60. doi: 10.1089/neur.2021.0080 (PMC9380885; doi:10.1089/neur.2021.0080)
Supplement: Supplemental data [file Supp_TableS8.docx]

Supplementary Table S8. Second computed tomographic findings in relation to outcome (27 young patients with acute subdural hematoma)

| Variable | Favorable | Unfavorable | p value | Alive | Dead | p value |
| --- | --- | --- | --- | --- | --- | --- |
|  | n = 16 | n = 11 |  | n = 22 | n = 5 |  |
| 2nd CT, day | 7 (6–7) | 7 (7–7) | 0.60 | 7 (6.75–7) | 7 (2.5–8) | 0.48 |
| Laterality, right, n (%) | 8 (50.0) | 5 (48.2) | 0.51 | 11 (50.0) | 2 (40.0) | 0.47 |
| Bilateral lesions, n (%) | 1 (6.3) | 6 (54.6) | **0.0049** | 4 (18.2) | 3 (60.0) | 0.054 |
| Contusion, n (%) | 11 (68.9) | 8 (72.7) | 0.82 | 17 (77.3) | 2 (40.0) | 0.99 |
| tSAH, n (%) | 1 (6.3) | 4 (36.4) | **0.048** | 3 (13.6) | 2 (40.0) | 0.17 |
| EDH, n (%) | 3 (18.8) | 0 (0) | 0.13 | 3 (13.6) | 0 (0) | 0.38 |
| SDH, n (%) | 3 (18.8) | 1 (9.1) | 0.49 | 4 (0) | 0 (0) | 0.30 |
| Thickness, mm | 0 (0–0) | 0 (0–0) | 0.50 | 0 (0–0) | 0 (0–0) | 0.31 |
| <5 mm, n (%) | 13 (81.3) | 10 (90.9) | 0.49 | 18 (81.8) | 5 (100) | 0.30 |
| ≥ 5, <10 mm, n (%) | 3 (18.8) | 1 (9.1) |  | 4 (18.2) | 0 (0) |  |
| ≥10 mm, n (%) | 0 (0) | 0 (0) |  | 0 (0) | 0 (0) |  |
| Midline shift, mm | 0 (0–2) | 4 (0–11) | **0.038** | 1 (0–2.25) | 10 (4–13) | **0.021** |
| <5 mm, n (%) | 9 (56.3) | 3 (27.3) | 0.12 | 11 (50.0) | 1 (20.0) | **0.020** |
| ≥5, <10 mm, n (%) | 5 (31.3) | 3 (27.3) |  | 8 (36.4) | 0 (0) |  |
| ≥10, <15 mm, n (%) | 2 (12.5) | 2 (18.2) |  | 2 (9.1) | 2 (40.0) |  |
| ≥15 mm, n (%) | 0 (0) | 3 (37.3) |  | 1 (4.6) | 2 (40.0) |  |
| Shift > thickness, n (%) | 6 (37.5) | 7 (63.6) | 0.18 | 9 (40.9) | 4 (80.0) | 0.11 |
| Basal cistern, n (%) |  |  |  |  |  |  |
| Normal, n (%) | 15 (93.8) | 7 (63.6) | 0.076 | 20 (90.9) | 2 (40.0) | **0.0006** |
| Compressed, n (%) | 1 (6.3) | 1 (9.1) |  | 2 (9.1) | 0 (0) |  |
| Absent, n (%) | 0 (0) | 3 (27.3) |  | 0 (0) | 3 (60.0) |  |
| Rotterdam Sum Score | 2 (2–2) | 3 (2–5) | **0.012** | 2 (2–2.25) | 5 (2–6) | **0.033** |
| 2, n (%) | 14 (87.5) | 5 (45.5) | 0.055 | 17 (72.3) | 2 (40.0) | **0.008** |
| 3, n (%) | 2 (12.5) | 2 (18.2) |  | 4 (18.2) | 0 (0) |  |
| 4, n (%) | 0 (0) | 0 (0) |  | 0 (0) | 0 (0) |  |
| 5, n (%) | 0 (0) | 2 (18.2) |  | 1 (4.6) | 1 (20.0) |  |
| 6, n (%) | 0 (0) | 2 (18.2) |  | 0 (0) | 2 (40.0) |  |
| ICP bleeding, n (%) | 1 (6.3) | 0 (0) | 0.40 | 1 (4.6) | 0 (0) | 0.63 |

tSAH, traumatic subarachnoid hemorrhage; EDH, epidural hematoma; SDH, subdural hematoma; ICP=intracranial pressure.

Values are presented as number (%) or median (interquartile range) unless otherwise indicated. Boldface type indicates statistical significance.
